# Supplementary material for: The Insulin-like Growth Factor Family as a Potential Peripheral Biomarker in Psychiatric Disorders: A Systematic Review
Source: Int J Mol Sci. 2025 Mar 12;26(6):2561. doi: 10.3390/ijms26062561 (PMC11942524; doi:10.3390/ijms26062561)
Supplement: Supplementary file 1 [file ijms-26-02561-s001.zip › ijms-3428934-supplementary.pdf]

Suplementary Materials

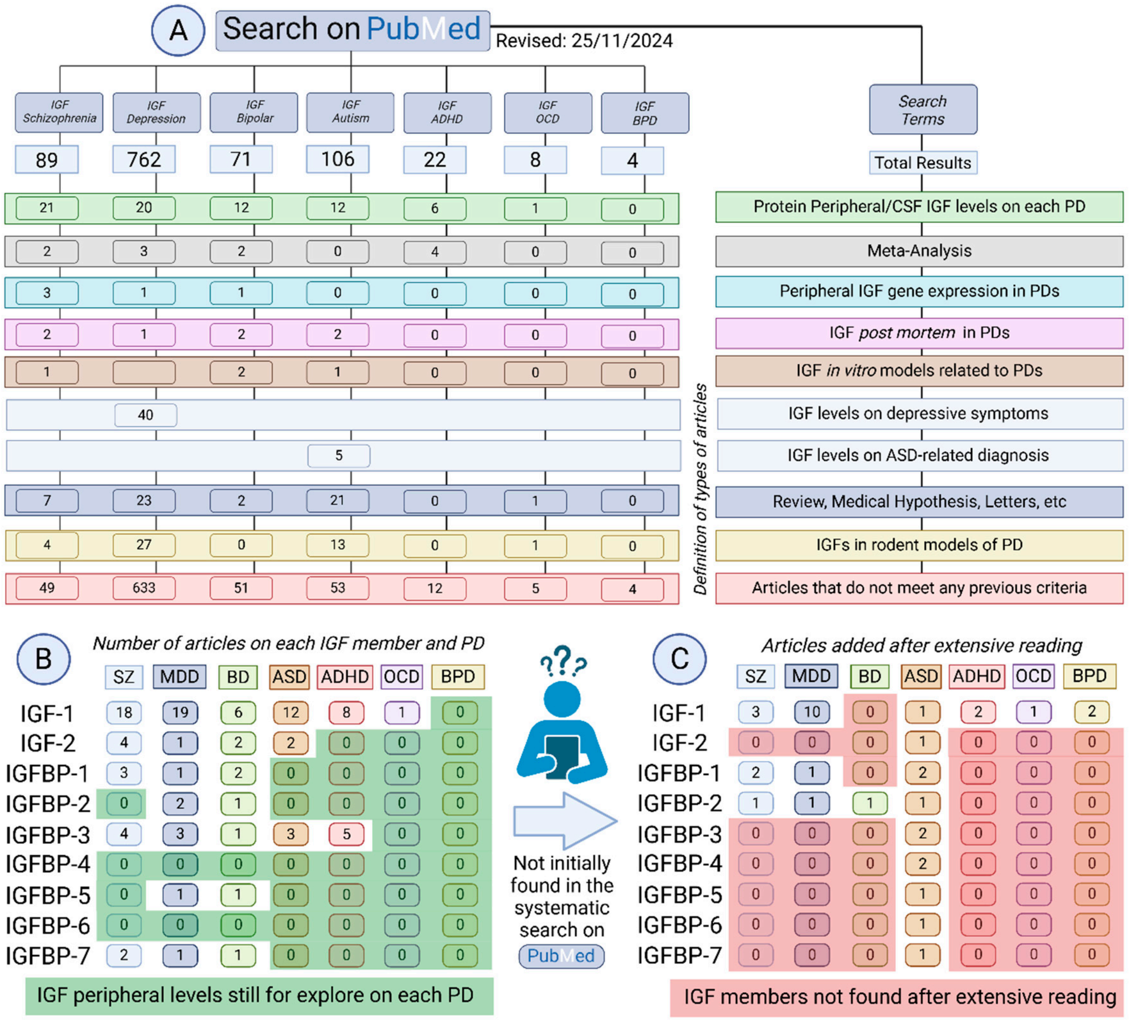

Figure S1. Detailed process of data collection: A) Shows the search terms and number of entries/results for each psychiatric disorder (PD) and type of article. We divided into ten different types of articles indicated by a colour legend. In green: original articles made in peripheral levels of IGF members with an official diagnosis of the PD. In gray: meta-analysis done from green articles. In blue: articles made in peripheral IGF gene expression. In violet: articles that measured IGFs in post-mortem samples on each PD. In brown: articles that worked on IGF levels on in cell lines derived from PDs (*in vitro* models). In light bluethese articles are exclusive for depression and ASD. In depression, because there are plenty studies (population-based, concomitant diseases with depressive symptoms, etc) that measured peripheral IGFs but without a proper diagnosis of MDD. This articles are commented but not included in Table 2 in the main text. In ASD, because they measured IGFs but in ASD-related diganosis such as McPhelan-Derman syndrome. In dark blue: non-original articles such as reviews, medical hypothesis, letters, etc. In yellow: articles made in rodent models of PDs and that includes IGFs. In red. Discarded articles that did not meet any previous criteria. B) Number of articles found on each member of the IGF family and PD in the first systematic search PubMed. C) Number of articles that were added after an extensive reading which were not found in the first systematic search. Highlighted in red are the IGF members for which no references were found after extensive reading. PubMed search ended at 25/11/2024. SZ (Schizophrenia), MDD (Major Depressive Disorder), BD (Bipolar Disorder), ASD (Autism Spectrum Disorder), ADHD (Attention Deficit Hyperactive Disorder), OCD (Obsessive Compulsive Disorder) and BPD (Borderline Personality Disorder). CSF (Cerebrospinal fluid). The number of articles may differ from Figure 2 in the main text, as duplicates are included in each search for each PD.

Table S1. Number or articles included in the main review of IGF in Schizophrenia (SZ).

**Reference**		**IGFs**		**IGFBPs**						
		1	2	1	2	3	4	5	6	7
**[35]**	Melkersson et al 1999	1	-	1	-	-	-	-	-	-
**[36]**	Melkersson et al 2000	1	-		-	-	-	-	-	-
**[37]**	Melkersson et al 2001	1	-	1	-	-	-	-	-	-
**[38]**	Melkersson et al 2003	1	-	1	-	-	-	-	-	-
**[39]**	Howes et al., 2004	1	-	1	-	-	-	-	-	-
**[44]**	Venkatasubramanian et al 2007	1	-		-	-	-	-	-	-
**[47]**	Wu et al 2007	1	-			1	-	-	-	-
**[87]**	Akanji et al 2007	1	1		1	1	-	-	-	-
**[55]**	Venkatasubramanian et al 2010	1	-	-	-	-	-	-	-	-
**[49]**	Yang et al 2012*	1	-	-	-	-	-	-	-	-
**[95]**	Schwarz et al 2012	-	-	-	1	-	-	-	-	-
**[73]**	Palomino et al 2013	1	-	-	-	-	-	-	-	-
**[50]**	Demirel et al 2014	1	-	-	-	-	-	-	-	-
**[93]**	Silva et al 2015	1	-	-	-	-	-	-	-	-
**[62]**	Petrikis et al 2016	1	-	-	-	-	-	-	-	-
**[67]**	Karanikas et al 2018	1	-	-	-	-	-	-	-	-
**[89]**	Chao et al 2020	1	-	-	-	-	-	-	-	-
**[88]**	Yang et al 2020	-	1	-	-	1	-	-	-	1
**[77]**	Chao et al 2020	-	1	-	-	-	-	-	-	-
**[69]**	Chen et al 2021	1								
**[83]**	Okamoto et al 2021	1	-	-	-	-	-	-	-	-
**[82]**	Yesilkaya et al 2021	1	-	-	-	-	-	-	-	-
**[92]**	Fernández-Pereira et al 2022	-	1	-	-	-	-	-	-	1
**[58]**	Arinami et al 2022	1	-	-	-	-	-	-	-	-
**[85]**	Pejcic et al 2023	Meta-analysis on IGF-1								
**[86]**	Xiong et al 2024	1	Meta-analysis on IGF-1							
	Total	21	4	4	2	3	0	0	0	2

The reference in the main manuscript is indicated in brackets. The name of the first author is also indicated with a colour mark. In black: the articles that were found in the first systematic search (Figure S1A and S1B). In red: the articles that were added after extensive reading (Figure S1C). In grey: meta-analysis. Xiong et al., 2024 [82] included both measures of IGF-1 and meta-analysis as it is indicated in the main text. The number of each cell represents the members of the IGF system measured in the article and gives a quick view of the number of IGF members studied in SZ. *Important note: the article of Yang et al 2012 [45] does not include measures of SZ patients, but their offspring. Nonetheless, we considered appropriate to include it in this group.

Table S2. Number or articles included in the main review of IGF in Major Depressive Disorder (MDD).

**Reference**		**IGFs**		**IGFBPs**						
		1	2	1	2	3	4	5	6	7
**[108]**	Lesch et al 1987	1	-	-	-	-	-	-	-	-
**[109]**	Lesch et al 1988	1	-	-	-	-	-	-	-	-
**[110]**	Lesch et al 1988	1	-	-	-	-	-	-	-	-
**[111]**	Lesch et al 1988	1	-	-	-	-	-	-	-	-
**[112]**	Lesch et al 1989	1	-	-	-	-	-	-	-	-
**[113]**	Lesch et al 1989	1	-	-	-	-	-	-	-	-
**[114]**	Rupprecht et al 1989	1	-	-	-	-	-	-	-	-
**[115]**	Lesch et al 1990	1	-	-	-	-	-	-	-	-
**[116]**	Brambilla et al 1994	1	-	-	-	-	-	-	-	-
**[113]**	Gann et al 1995	1	-	-	-	-	-	-	-	-
**[132]**	Michelson et al 1996	1	-	-	-	-	-	-	-	-
**[118]**	Deuschle et al 1997	1	-	-	1	1	-	-	-	-
**[133]**	Franz et al 1999	1	-	-	-	-	-	-	-	-
**[142]**	Michelson et al 2000	1	-	-	-	-	-	-	-	-
**[119]**	Weber-Hamann et al 2009	1	-	-	-	-	-	-	-	-
**[143]**	Stelzhammer et al 2013	1	-	-	-	-	-	-	-	-
**[135]**	Li et al 2013	1	-	-	-	-	-	-	-	-
**[146]**	Krogh et al 2014*	1	-	-	-	1	-	-	-	-
**[120]**	Kopzack et al 2014	1	-	-	-	-	-	-	-	-
**[136]**	Tu et al 2016	Meta-analysis on IGF-1								
**[122]**	Bot et al 2016	1	-	-	-	-	-	-	-	-
**[205]**	Lamers et al 2016	-	-	1	1	-	-	-	-	-
**[124]**	Rosso et al 2016	1	-	-	-	-	-	-	-	-
**[206]**	Milanesi et al 2018	-	-	-	1	-	-	-	-	-
**[121]**	Tajiri et al 2019	1	-	-	-	-	-	-	-	-
**[126]**	Levada 2020	1	-	-	-	-	-	-	-	-
**[127]**	Troyan and Levada 2020	1	-	-	-	-	-	-	-	-
**[140]**	Shi et al 2020	Meta-analysis on IGF-1								
**[139]**	Chen et al 2020	Meta-analysis on IGF-1								
**[128]**	Ali et al 2020	1	-	-	-	-	-	-	-	-
**[129]**	Arinami et al 2021	1	-	-	-	-	-	-	-	-
**[130]**	Arinami et al 2023	1	-	-	-	-	-	-	-	-
**[193]**	Fernández-Pereira et al 2023	-	1	1	-	1	-	1	-	1
**[130]**	Arinami et al 2024	1	-	-	-	-	-	-	-	-
**[131]**	Qiao et al 2024	1	Meta-analysis on IGF-1							
	Total	29	1	2	3	3	0	1	0	1

The reference in the main manuscript is indicated in brackets. The name of the first author is also indicated with a colour mark. In black: the articles that were found in the first systematic search (Figure S1A and S1B). In red: the articles that were added after extensive reading (Figure S1C). In grey: meta-analysis. Qiao et al 2024 [128] included both measures of IGF-1 and meta-analysis as it is indicated in the main text. The number of each cell represents the members of the IGF system measured in the article and gives a quick view of the number of IGF members studied in MDD. *Important note: the article of Krogh et al 2014 [144] is the only one included in Table 2 in the main text because it involved patients with an official diagnosis of MDD.

Table S3. Number or articles included in the main review of IGF in Bipolar Disorder (BD).

Reference		IGFs		IGFBPs						
		1	2	1	2	3	4	5	6	7
**[217]**	McIntyre et al., 2003	1	-	1	-	-	-	-	-	-
**[222]**	Kim et al., 2013	1	-	-	-	-	-	-	-	-
**[73]**	Palomino et al 2013	1	-	-	-	-	-	-	-	-
**[224]**	Liu et al., 2014	1	-	-	-	-	-	-	-	-
**[242]**	Benedetti et al., 2016	-	-	-	1	-	-	-	-	-
**[136]**	Tu et al., 2016	Meta-analysis on IGF-1								
**[231]**	da Silva et al., 2017	1	-	-	-	-	-	-	-	-
**[206]**	Milanesi et al., 2018	-	-	-	1	-	-	-	-	-
**[233]**	Ferensztajn-Rochowiak et al., 2019	1	-	-	-	-	-	-	-	-
**[225]**	Tunçel et al., 2020	1	-	-	-	-	-	-	-	-
**[139]**	Chen et al., 2020	Meta-analysis on IGF-1								
**[234]**	Guldiken et al., 2024	1	-	-	-	-	-	-	-	-
**[236]**	Fernández-Pereira et al., 2024	-	1	1		1		1	-	1
**[235]**	Ye et al., 2024	-	1	-	-	-	-	-	-	-
	Total	8	2	2	2	1	0	1	0	1

The reference in the main manuscript is indicated in brackets. The name of the first author is also indicated with a colour mark. In black: the articles that were found in the first systematic search (Figure S1A and S1B). In red: the articles that were added after extensive reading (Figure S1C). In grey: meta-analysis. The number of each cell represents the members of the IGF system measured in the article and gives a quick view of the number of IGF members studied in BD.

Table S4. Number or articles included in the main review of IGF in Obsessive Compulsive Disorder (OCD) and Borderline Personality Disorder (BPD)

Reference		IGFs		IGFBPs						
		1	2	1	2	3	4	5	6	7
**[246]**	Rosmond et al 1999	1	-	-	-	-	-	-	-	-
**[245]**	Kahl et al 2005	1	-	-	-	-	-	-	-	-
**[124]**	Rosso et al 2016	1	-	-	-	-	-	-	-	-
**[251]**	Narayanaswamy et al 2017	1	-	-	-	-	-	-	-	-
	Total	4	0	0	0	0	0	0	0	0

The reference in the main manuscript is indicated in brackets. The name of the first author is also indicated with a colour mark. In black: the articles that were found in the first systematic search (Figure S1A and S1B). In red: the articles that were added after extensive reading (Figure S1C). The number of each cell represents the members of the IGF system measured in the article and gives a quick view of the number of IGF members studied in OCD and BPD.

Table S5. Number or articles included in the main review of IGF in Autism Spectrum Disorder (ASD).

Reference		IGFs		IGFBPs						
		1	2	1	2	3	4	5	6	7
**[257]**	Vanhala et al., 2001	1	-	-	-	-	-	-	-	-
**[25]**	Vargas et al., 2005	-	-	1	-	1	1	-	-	-
**[259]**	Riikonen et al., 2006	1	1	-	-	-	-	-	-	-
**[262]**	Mills et al., 2007	1	1	-	-	1	-	-	-	-
**[263]**	Anlar et al., 2007	1	-	-	-	1	-	-	-	-
**[261]**	Makkonen et al ., 2011	1	-	-	-	-	-	-	-	-
**[264]**	Neumeyer et al., 2017	1	-	-	-	-	-	-	-	-
**[265]**	Simsek et al., 2021	1	-	-	-	-	-	-	-	-
**[267]**	Robinson-Agramonte et al., 2021	1	-	-	-	-	-	-	-	-
**[268]**	Abedini et al., 2022	1	-	-	-	-	-	-	-	-
**[266]**	Robinson-Agramonte et al., 2022	1	-	-	-	-	-	-	-	-
**[269]**	Li et al., 2022	1	-	-	-	1	-	-	-	-
**[26]**	Mashayekhi et al., 2022	1	1	1	1	1	1	1	1	1
**[234]**	Guldiken et al., 2024	1	-	-	-	-	-	-	-	-
	Total	13	3	2	1	5	2	1	1	1

The reference in the main manuscript is indicated in brackets. The name of the first author is also indicated with a colour mark. In black: the articles that were found in the first systematic search (Figure S1A and S1B). In red: the articles that were added after extensive reading (Figure S1C). The number of each cell represents the members of the IGF system measured in the article and gives a quick view of the number of IGF members studied in ASD.

Table S6. Number or articles included in the main review of IGF in Attention-Deficit Hyperactive Disorder (ADHD)

Reference		IGFs		IGFBPs						
		1	2	1	2	3	4	5	6	7
**[293]**	Schultz et al., 1984	1	-	-	-	-	-	-	-	-
**[294]**	Toren et al., 1997	1	-	-	-	-	-	-	-	-
**[295]**	Bereket et al., 2005	1	-	-	-	1	-	-	-	-
**[296]**	Kim et al., 2020	1	-	-	-	-	-	-	-	-
**[299]**	Mei et al., 2022	1	-	-	-	-	-	-	-	-
**[300]**	Wang et al., 2022	1	-	-	-	1	-	-	-	-
**[301]**	Wang et al., 2023	1	-	-	-	1	-	-	-	-
**[302]**	Etrük et al., 2024	1	-	-	-	-	-	-	-	-
**[303]**	Van Andel et al., 2024	1	-	-	-	-	-	-	-	-
**[297]**	Velayutham et al., 2024	1	-	-	-	1	-	-	-	-
	Total	10	0	0	0	4	0	0	0	0

The reference in the main manuscript is indicated in brackets. The name of the first author is also indicated with a colour mark. In black: the articles that were found in the first systematic search (Figure S1A and S1B). In red: the articles that were added after extensive reading (Figure S1C). The number of each cell represents the members of the IGF system measured in the article and gives a quick view of the number of IGF members studied in ADHD.
